# M. Bally on the Therapeutic Effects of Morphine
*Mémoires de l'Acad. Roy. de Médecine, tome 1.


**Published:** 1830-04-01

**Authors:** 


### XLVIII.

### M. Bally on the Therapeutic Effects of Morphine.*

We have reason to know, from observation at the bedside, that morphine is a very powerful and often a very excellent narcotic or sedative agent. But there are cases in which it fails, and there are cases in which it disagrees, so that it becomes an object to discriminate the one and ascertain the other, as far as possible, by clinical experiments. We are induced, on these accounts, to notice a valuable, but prolix, memoir on the effects of morphine, by M. Bally; and although in our hands it will be somewhat shorn of its fair proportions, we believe that the residuary *extractive* will not be entirely inefficacious.

M. Bally calculates, that if, when prepared in the best manner, 16 ounces of the solution of opium furnish $8\frac{1}{2}$ *gros*, the opium ought to contain $38\frac{1}{4}$ grains per ounce, or about 4 grains per *gros*, which constitutes a little more than one-sixteenth.

Various experiments have been made at the College of Alfort, by M. Dupuy and M. Lassaigne, in order to ascertain how far acetate of morphine may be detected by chemical or other tests in case of poisoning. As this is rather a subject for the chemist than the medical practitioner, we shall content ourselves with mentioning the broad conclusions to which the said experiments lead. First, it is possible, in many instances, to detect, by chemical means, sensible traces of this vegetable poison; secondly, it is always in the viscera where the poison was originally introduced, that such traces of its presence are discovered; thirdly, the matters vomited in a short time after the reception of the poison into the stomach, are found to contain ponderable quantities of it; fourthly, all efforts to discover it in the blood have been ineffectual. Morphine is usually administered, in combination with acetic acid, in the form of acetate, from the belief that its operation is thus rendered more certain and its activity increased. M. Bally, however, has been led by actual experiments to the conclusion, that this is not the fact, and that the salifiable base, or pure morphia, is equal in every respect to the acetate, and may always be substituted for it. If morphia is given it should be in pills, on account of its insolubility in fluid vehicles, and, indeed, this mode of administration is preferred by our author on several accounts. He thinks it advisable, however, to vary the mode of exhibition occasionally, giving sometimes the acetate or sulphate in solution, sometimes the morphia in pill, and sometimes half a grain or a grain in lavement. M. Bally usually exhibits a quarter of a grain twice daily to men, and about half that quantity to women, augmenting or graduating the dose, according to its effects upon the system. To the consideration of those effects we now proceed. Sometimes a sensation of bitterness is experienced in the mouth, and M. Bally declares this to be a forerunner of vomiting, unless the exhibition of the remedy is desisted from. A woman, ætatis 50, who was taking five quarters of a grain of the morphine per diem, for rheumatic pains, began to be affected with bitterness in the mouth. She continued the remedy till she arrived at seven quarters of a grain daily, when violent vomiting of green matters supervened, with much anguish and great hardness of the pulse. The medicine was of course abandoned, but much epigastric tenderness remained long afterwards. Given in a dose, varying according to the patient's idiosyncrasy, morphine excites violent and prolonged vomiting. A less quantity suffices with women than with men, but the abrupt exhibition of a grain to the latter will commonly operate in this manner. The nausea or vomiting commonly occur very shortly after the reception of the medicine in the stomach, and our author has seen females, in whom the eighth of a grain given twice a day produced a state of permanent nausea, or distressing vomiting.

Another symptom produced by the

* Mémoires de l'Acad. Roy. de Médecine, tome 1.

morphine is the extrication of gas in the stomach, a symptom that is always the prelude of nausea. With respect to its action on the intestinal canal, it must be observed that it generally constipates in the first instance, but frequently copious diarrhœa succeeds, and colicky pains are experienced in the vicinity of the umbilicus. M. Bally thinks the salt is possessed of some vermifuge powers, for in one case ascarides lumbricoides were voided during its exhibition, and in another five worms were thrown up by vomiting.

M. Bally believes that the salts of morphia have no action on the kidneys, but nineteen-twentieths of the patients who take it experience some difficulty in making water, amounting in some to complete retention, a symptom which disappears with the removal of its cause. It is remarkable that females are never affected in this manner, but our author has known two or three complain of a sense of heat in micturition. The colour of the urine is never altered.

Morphine never stimulates the heart nor excites the pulse, the latter being rarely higher than from sixty-five to eighty in the minute, and not unfrequently falling lower during its administration. M. Bally observes that morphine, having little or no effect on the thoracic viscera, seldom or never allays that troublesome symptom, cough. If it seems to do so the amendment is delusive, for a day or two dispels the agreeable deception. We have ourselves observed this circumstance, having seen the morphine fail completely in allaying the cough that remains after acute attacks of pleurisy. Morphia is not at all sudorific, but frequently produces an intolerable pruritus or itching on the skin, unattended by cutaneous redness or inflammation. Sometimes the itching is general, more frequently partial, and, in three cases, it extended to the sexual organs of the female, and especially invaded the penis of the male.

In some subjects, the pruritus was accompanied by an eruption of small conical papillæ, sometimes red, sometimes without any decided colour. These symptoms seldom appear during the first days of the administration of the remedy, and pass away when it is discontinued, or even whilst its exhibition is persisted in.

Although morphine or its salts have no exciting effect on the heart and arteries, M. Bally thinks there can be little doubt that it stimulates the brain, and even favours the occurrence of apoplexy and extravasations of blood within the cranium. In proof of this he cites some curious facts.

*Case.* Jean Marchand, æt. 60, of apoplectic make and figure, had an attack of the disease in 1809, which ended in hemiplegia of the left side. In 1823, the patient entered the hospital to which M. Bally is attached, being then incapable of supporting himself on the left lower extremity, and suffering from a considerable contraction of the arm; his articulation was indistinct, and his intellectual faculties impaired. After some bleedings, ice was applied to the head, and acetate of morphine exhibited in the dose of a quarter of a grain night and morning. When the patient had arrived at seven quarters of a grain in the day, he was attacked with insomnia, head-ache and delirium, for which he was immediately bled, and the morphine discontinued. Under these measures the alarming symptoms disappeared, and, by the next day, Marchand was in his usual health. Twelve days after this explosion of bad symptoms, M. Bally determined to make another experiment with the acetate, omitting the application of ice to the head. A quarter of a grain was administered morning and evening, but, on the third day, the pulse became hard, full and frequent, the tongue was dry, and the patient delirious in the night. Two bleedings were practised, after which the delirium became furious, and, on the fifth day, consciousness was totally lost. The thoracic organs were "embarrassed," expectoration ceased, the tongue was dry and red, the pulse frequent, and the patient uttered cries at intervals. On the 6th day after resuming the morphine, and the third from the commencement of the bad symptoms he died.

*Sectio Cadaveris.* The skin was not discoloured. Between the arachnoid and pia mater was a pretty fair quantity of clear serous fluid, without any injection of the membranes. In the posterior part of the left hemisphere of the brain was a recent extravasation of blood, which had forced itself into three of the convolutions, and produced considerable ramollissement of the cerebral matter. The left optic thalamus was marked, like old cheese, with several small *foyers*, "which, no doubt, had been the seats of old extravasations of blood." At the posterior and superior part of the right corpus striatum, was a depression large enough to contain a French-bean, lined with a fine false membrane, and considered by our author to have been the seat of the old apoplectic extravasation, which produced the hemiplegia of the left side. The arteries of the brain were in a morbid state, and presented many cartilaginous points in their tunics. The stomach was of a remarkably blue colour towards its lesser curvature, but nothing further was observed in the thorax or abdomen.

M. Bally has no doubt whatever that the exhibition of the morphine was the cause of death, by producing the recent extravasation in the brain. In two other cases of hemiplegia and palsy related by our author the morphine exasperated the cerebral symptoms, and was abandoned in consequence. These facts should put physicians on their guard.

Considering a narcotic as something more than merely a medicine producing sleep, morphine is decidedly to be considered as such. In some it produces a state like intoxication, in others vertigo, and in others again sensations like electric shocks. Some patients are affected with strange hallucinations, hearing unreal noises and seeing unreal sights. A very common effect is a dimness of vision, amounting at times to momentary incapabability of reading, and from the result of much experience and the observation of numerous cases, M. Bally does not hesitate to say that the slow and successive action of the salts of morphine induces *contraction* of the pupils. But it is a curious fact that as soon as this contraction has passed away the pupils have a tendency to dilatation. Of this fact many examples are cited by M. Bally to which we need only allude. In three cases only has M. Bally witnessed primary dilatation of the pupil, and he enters into a discussion to prove that contraction of the pupils is in general the accompaniment of poisoning by opium. In some cases of deep intoxication we have observed this state of the pupil, and in the instance of a man affected with delirium tremens, who took very large quantities of opium, we noticed the same thing. M. Bally mentions a curious circumstance that occurred to him:—eleven hemiplegiac patients in one ward being given the morphine for some length of time, the pupils in all became contracted; but the plan not producing any good effects they were all put upon strychnine, and very shortly the pupils in all became dilated! Our author, however, is inclined to believe that, when given in the form of enema, morphine has a tendency to dilate the pupils; if combined with a remedy whose effects upon the iris are opposed to its own, the pupil is acted on by neither.

The effects of morphine in producing sleep are very variable, but generally small doses answer better for this purpose than large ones, and what is singular, the season appears to have a perceptible effect on this property of the drug. It does not always, but it does occasionally excite head-ache, generally preceded by vertigo, and more frequently produced in women than in men.

In the production of sleep and their other effects it would be very difficult to ascertain with any accuracy the relative powers of opium, its extracts, and morphine. If any positive proportion existed, a fifteenth of a grain of morphine *should* be equal to one of crude opium, the latter being to the former as 15 to 1. Those, however, who have exhibited morphine will hardly find its actual powers so great as these speculative numbers imply: indeed M. Bally thinks it probable, that a grain of the aqueous extract of opium is more narcotic than a quarter of a grain of morphine.

M. Bally closes his memoir by alluding to the endermic method of administering this and other remedies. He has employed it with very great success, for of two men affected with paralysis of the hands one was evidently cured by the application of a grain to a grain and a half of strychnine daily to the raw surface produced by a blister; the second recovered the use of one hand and nearly recovered that of the other; and a third paraplegiac patient is beginning to walk. Morphine applied in this manner produces "marvellous effects" on rheumatisms, lumbago, and sciatica; and so far do the powers of this remedy extend, that even the indomitable tetanus has been thought to succumb beneath them. Our author has received the histories of three cases of tetanus so treated with success; one was traumatic, the second produced by strychnine, and the third by a fright. Alas! we fear that tetanus is not yet to bow to a mightier than morphine!

This concludes M. Bally's memoir, and we are bound to declare that it evinces a patient and persevering industry of observation and experiment that redound vastly to his credit as a hospital physician. If our neighbours follow so excellent an example they will soon wipe away the stigma of *expectantism* with which their practice is at present branded, we fear too justly, and will lead the van in the appreciation of the powers of medicines, as they have done for some time in the cultivation of morbid anatomy. En avant, say we, and we shall regard with no jealousy the advance of the Eagle of Gallic Science, even though it flap its wings before what Napoleon tauntingly denominated the Leopard of England.

